# Preface to ‘Modelling of dynamic phenomena and localization in structured media’ in honour of Professor Leonid Slepyan on the occasion of his 90th birthday

**DOI:** 10.1098/rsta.2019.0134

**Published:** 2019-09-02

**Authors:** G. Mishuris, A. Movchan, L. Truskinovsky

**Affiliations:** 1 Department of Mathematics, Institute of Mathematics, Physics and Computer Science, Aberystwyth University, Aberystwyth, Wales, UK; 2 Department of Mathematical Sciences, University of Liverpool, Liverpool, UK; 3 Laboratoire de Physique et Mécanique des Milieux Hétérogénes (PMMH UMR 7636) CNRS, ESPCI Paris, PSL Research University, 10 rue Vauquelin, 75005 Paris, France

We are honoured to dedicate this issue of Philosophical Transactions of the Royal Society to Leonid Slepyan, Professor Emeritus of the Tel Aviv University, who has recently celebrated his 90th birthday.


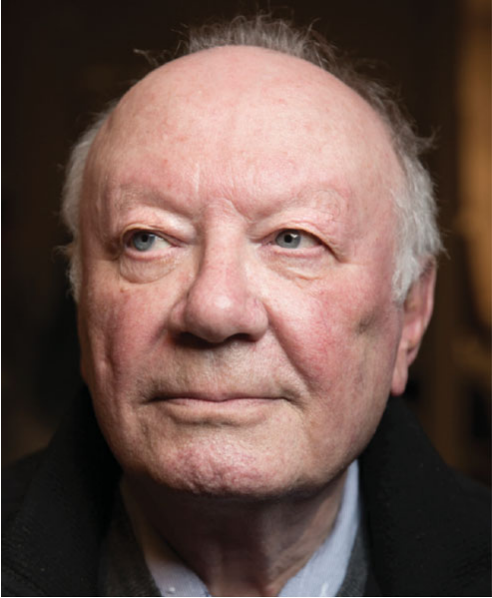



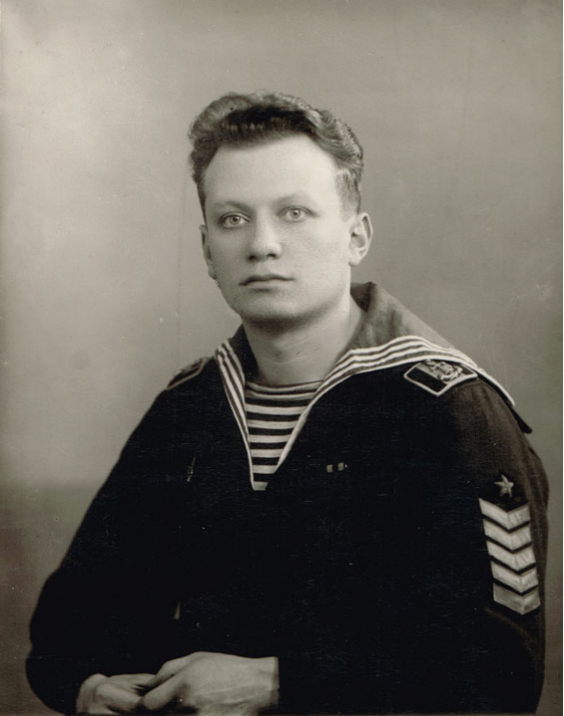


Leonid Slepyan's work on the dynamics of structured media, in particular, his classical theory of elastic lattices with moving cracks, stands out as a truly pioneering achievement that has laid the foundations for several new directions in the mathematical modelling of inhomogeneous solids. Leonid Slepyan is a great Master who created an influential school of dynamic fracture, which is now actively extending and deepening his ideas. The broader impact of Leonid Slepyan's work on the community of solid mechanicians cannot be overestimated.

While Leonid Slepyan is mostly known for his fundamental results in theoretical mechanics, he started his professional career as a naval engineer. After graduation from the Naval Academy Engineering School in St Petersburg (at that time Leningrad, USSR) in 1951, he worked for 6 years on ship repair plants. One of the technical operations for which he was responsible became the subject of his first publication. This first paper was published in 1957, in which he introduced an unexpected but very effective procedure for the partial docking of a vessel with a small floating dock, allowing, for instance, an urgent repair of a submarine. The description of this novel methodology is still integrated as a part of the technical syllabus in St Petersburg Marine Technical University.

Then, as a senior researcher in a Naval Research Institute, Leonid Slepyan worked on practical problems, like the design of floating and dry docks and the stability of naval structures under pressure waves. This applied work led to remarkable theoretical generalizations related to the vibration of submerged bodies, waves under moving loads, resonant wave phenomena and mechanical perforation. Most of these studies were summarized in the prize-winning research monograph ‘Non-stationary elastic waves’.

Later Leonid Slepyan moved to the Siberian Branch of the USSR Academy of Sciences, and eventually became a Professor and a Head of Department of the Marine Technical University in St Petersburg Russia. While working there, he continued to combine fundamental theoretical work with a flow of spectacular inventions in the domain of applied engineering. Starting from 1992, Leonid Slepyan held the position of Professor (now Professor Emeritus) at the School of Mechanical Engineering at Tel Aviv University.


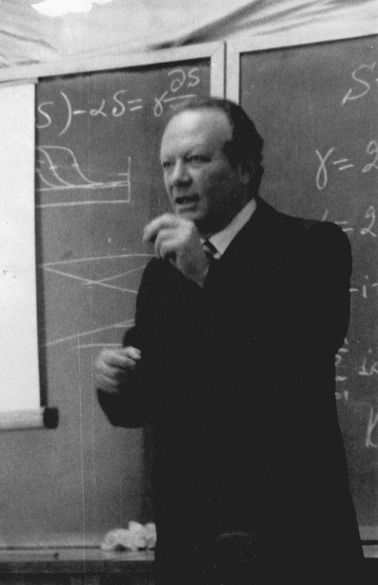


In appreciation of his outstanding contributions to the field, in 2014 Leonid Slepyan was named a senior Marie-Curie Professor. This prestigious award allowed him to spend a considerable amount of time working with different groups in the UK. In this extremely fruitful period, Leonid Slepyan obtained some of his fundamental results in the new field of metamaterials.

Leonid Slepyan's approach has always been truly original and his methods are always fundamental. A characteristic of his scientific philosophy is to find new results under most general conditions and his motto can be formulated as: ‘generality leads to transparency and simplicity’. Also characteristic of Leonid Slepyan's approach is to use unexpected and exquisitely executed asymptotic approximations that are always fully analytical.

As an example, we mention Leonid Slepyan's solutions to the dynamic problems of fracture in elastic–plastic bodies that are well known to specialists. He was the first to find an elegant explanation for the dramatic difference between the intensity of the stress field singularity for a quasi-statically and dynamically advancing crack when compared with that of a static crack. One can say that Leonid Slepyan's seminal papers on this subject opened a completely new chapter in the literature on fracture mechanics. In a paper with the deceptively simple title ‘Crack Dynamics in a Lattice’, he used sophisticated methods of analysis to derive an analytical relation between the macroscopic energy release and the crack velocity. Most strikingly, starting with a dissipation-free system, he managed to compute the energy flux from ‘macro’ to ‘micro’ scale and in this way, he was able to determine the apparent dissipative properties of a Hamiltonian crack. This paper paved the way for a large number of studies by Leonid Slepyan and his followers, who extended this approach to the description of general dynamic defects (cracks, dislocations, phase boundaries, etc.) in structured media. All this body of work hinges on the brilliant theoretical insight of Leonid Slepyan: that in this class of problems, the phenomena at continuum level cannot be treated as independent of the processes at the scale of the microstructure.

Leonid Slepyan's more recent work at Tel Aviv University has continued to be extremely innovative and stimulated several new research directions, including the analysis of dynamic phase transitions, failure waves, dynamic nucleation in elastic lattice systems, dynamic Green's kernels, etc. His related work on dynamic homogenization revealed new perspectives in the study of stop bands and exponential localization. During this period, Leonid Slepyan published his famous monograph ‘Models and Phenomena in Fracture Mechanics’, which is now considered as a kind of Bible by all researchers working in the area of wave dispersion and dynamic defects in structured solids. It contains a description of many unsolved problems and, with its deep insights, has inspired a whole generation of young scientists. For example, a reference to the book associated with the term ‘Slepyan's crack’ is nowadays commonly used in the research literature to indicate dispersive energy propagation and localization in the dynamics of structured solids.

In the last few years, Leonid Slepyan's ideas have found some dazzling applications in the new field of dynamic metamaterials, where micro-structural effects are particularly important. It is enough to mention Slepyan's stunning work on solitons in helical structures, his discovery of complex fracture modes for binary cracks in lattices and his highly original exploratory study of clustering and forerunning regimes in elastodynamics.

Among the most recent results, it is important to highlight Leonid Slepyan's truly fundamental paper, resolving the problem of the energy partition in waves formulated 138 years ago by Lord Rayleigh! Along the way (and using the same ‘first principles’ approach), Leonid Slepyan introduced the new concept of ‘wave mass’ and finally clarified some intriguing and much debated aspects of the notion of wave momentum.

It is, of course, impossible in such a short introductory article to review in depth all the ground-breaking results of Leonid Slepyan. A bigger picture emerges from the technical papers collected in this Theme Volume. It is published in two volumes, which reflects the overwhelming response of the international mechanics and mathematics community to Prof. Slepyan's 90th birthday. The complete list of Leonid Slepyan's publications is available on the web page: https://www.eng.tau.ac.il/~leonid/publications.html.


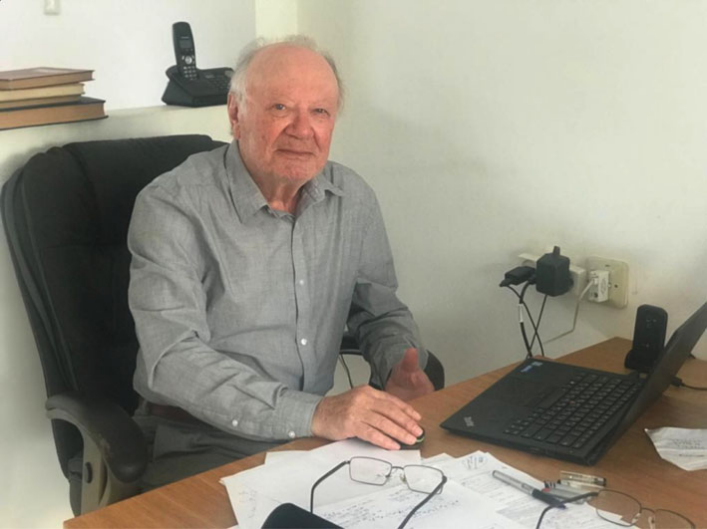


While being a profound thinker with highly influential ideas, Leonid Slepyan is known to his colleagues as a very gentle person with admirable humility. His impeccable human ethics make a very strong and lasting impression on anyone who comes into contact with him.

Reaching a respectable 90th birthday, Leonid Slepyan is not slowing down, producing each year a steady flow of outstanding papers. His creativity and technical mastery have served as an inspiring example for generations of researchers in the fields of Mechanics and Applied Mathematics and his influence on the hearts and minds of young researchers continues to spread. We use this opportunity to express our sincere gratitude to Leonid Slepyan, whom we consider our teacher in both science and life. On the occasion of his 90th birthday, we wish him many more years of strong health and wonderful research results.

